# Strategies for precision modulation of gene expression by epigenome editing: an overview

**DOI:** 10.1186/s13072-015-0023-7

**Published:** 2015-09-17

**Authors:** Benjamin I. Laufer, Shiva M. Singh

**Affiliations:** Molecular Genetics Unit, Department of Biology, University of Western Ontario, London, ON Canada

**Keywords:** Regulation of gene expression, Functional genomics, Stem cells, dCas9, CRISPR/Cas9, Zinc Finger, Transcription-Activator-Like Effector (TALE), Synthetic biology

## Abstract

Genome editing technology has evolved rather quickly and become accessible to most researchers. It has resulted in far reaching implications and a number of novel designer systems including epigenome editing. Epigenome editing utilizes a combination of nuclease-null genome editing systems and effector domains to modulate gene expression. In particular, Zinc Finger, Transcription-Activator-Like Effector, and CRISPR/Cas9 have emerged as modular systems that can be modified to allow for precision manipulation of epigenetic marks without altering underlying DNA sequence. This review contains a comprehensive catalog of effector domains that can be used with components of genome editing systems to achieve epigenome editing. Ultimately, the evidence-based design of epigenome editing offers a novel improvement to the limited attenuation strategies. There is much potential for editing and/or correcting gene expression in somatic cells toward a new era of functional genomics and personalized medicine.

## Background

The modulation of gene expression can be achieved by a variety of biotechnologies such as RNA interference, non-precision drugs, and artificial transcription factors (ATFs). Epigenome editing is an emerging field of synthetic biology that falls under the category of ATF [1]. It is distinguished from other gene expression modulation technologies in that it can create precise and long-lasting epigenetic modification without the need to keep or maintain the system after the initial event [2].

The epigenome editing systems that are the focus of this review contain the DNA-binding element of genome editing systems. Thus, in order to gain a full appreciation for epigenome editing one must start with the fundamentals of genome editing as the two share not only components but also obstacles. Genome editing represents a revolution in genetic engineering as it allows for precision targeting and manipulation of genome. Genome editing systems rely on two components, a DNA-binding element, and nuclease, to modify the targeted DNA sequence. Genome editing can be used to study protein function by altering coding sequence or achieve transcriptional control by altering the sequence of regulatory regions. Epigenome editing, on the other hand, uses the same DNA-binding principle but utilizes an effector domain, rather than a nuclease. The effector domain is a fragment of a desired regulatory protein and is used to create a desired epigenetic mark at a targeted locus without altering the underlying sequence.

### DNA-binding genome editing systems

The concept of genome editing is not new. What is new is the refinement of methods that make it feasible for most laboratories to undertake the protocol successfully. Today, genome editing allows for precise manipulation of DNA sequences and brings about desired genetic changes at will in vitro and in vivo [3–5]. The principle involves precise targeting of a specific DNA sequence in the genome to create a site-specific double-stranded break using a nuclease. A cell will then attempt to correct this damage by homology-directed repair (HDR), which makes it possible to introduce desired donor sequence(s). Additionally, non-homologous end-joining (NHEJ) can be used to delete desired sequences. The methods available make use of Zinc Fingers (ZFs), Transcription-Activator-Like Effectors (TALEs), and the Clustered Regulatory Interspaced Short Palindromic Repeats (CRISPR) sequences with CRISPR-Associated Protein 9 (Cas9) detailed below.

#### ZFN

**Fig. 1:**
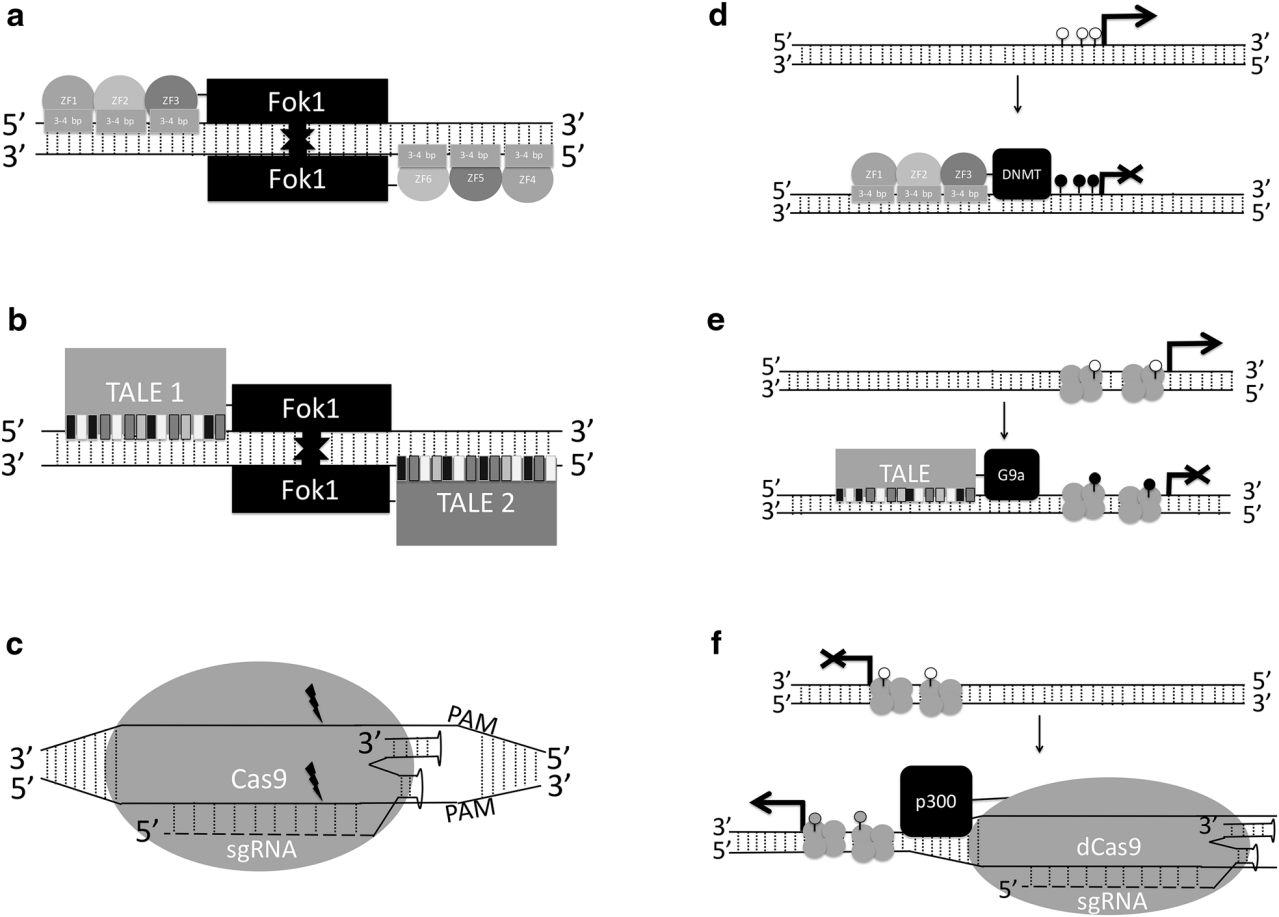
(Epi)Genome editing systems. **a** Zinc Finger Nuclease (ZFN), **b** Transcription-Activator-Like Effector Nuclease (TALEN), **c** CRISPR/Cas9, **d** Zinc Finger (ZF) with a DNA methyltransferase effector domain, **e** Transcription-Activator-Like Effector (TALE) with a histone methyltransferase, and **f** Catalytically deactivated Cas9 (dCas9) and sgRNA from with a histone acetyltransferase. Components are not to scale as critical features are exaggerated and some non-variable features are removed

Zinc Finger Nuclease (ZFN) is the oldest genome editing technology [6–8]. It is based on two-modules. The first are Zinc Finger Proteins that recognize and bind to DNA sequences. Zinc finger proteins coordinate zinc ions using a backbone of conjugated Cysteine (Cys) and Histidine (His) residues to achieve their structure. They come in a number of folding groups the most widely used being the Cys_2_His_2_ group. This folding group represents the classical zinc finger and is widely used as a natural transcription factor in mammals. Cys_2_His_2_ ZFs also have a relatively conserved backbone. ZF specificity for DNA sequence comes from a part of the α helix, known as the recognition domain, which binds to the major groove of DNA. The specificity is determined by amino acids in the recognition domain. Variation in this region, either naturally occurring or synthetic, results in recognition of alternative nucleic acid sequences. Experimentally, a designer zinc finger is fused to a nuclease (*Fok*I), which requires dimerization for double-stranded DNA cleavage. Here, the targeting specificity comes about from the recognition domain, with each ZF recognizing 3–4 bp per amino acid in the domain. Typically, ZFN systems use a combination of 3–6 ZFs fused to a *FokI* domain. The inverted dimer required for nuclease activity gives additional sequence specificity as there is a required space, known as the spacer. This design approach facilitates a target specificity of ~24 bp, which is enough to target most unique regions in most genomes (Fig. 1a). In terms of practicality, ZFNs are limited by the higher cost and effort of designing the custom proteins, interactions between residues affecting targeting, and altered sequence recognition from the effects of additional genomic and chromatin content surrounding the target sites [9]. However, they have the advantage of being oldest, most studied, and only genome editing system to be in clinical trials.

#### TALEN

TALENs represent a fusion of a Transcription-Activator-Like Effector (TALE), which is a viral element evolved to target plant transcription and a designer nuclease [10–12]. TALEs have a central repeat domain that confers its sequence specificity (Fig. 1b). This domain is 33–35 amino acids long and has two highly variable sites at the 12th and 13th amino acids, which are known as the repeat variable di-residues (RVDs). Different combinations at the RVDs allow for recognition of a single base. While both ZFN and TALEN systems theoretically allow for single-base targeted design, TALEs recognize single nucleotides as opposed to the triplet combinations of a ZFPs recognition sequence. Also, this system performs better than ZFNs since they are not as influenced by sequence and chromatin surrounding the target site [13–17]. More importantly, TALEs and TALENs represent a significant improvement in specificity and protocol [18, 19].

TALENs have been successfully used in mice for mitochondrial transfer, which allows for a 3-parent child. This is done in order to prevent an inherited disorder in the mitochondrial genome that would normally be transmitted from the mother. It has recently been approved as a medical procedure in the UK and is currently under serious consideration in USA. Intriguingly, a mitoTALEN system was recently used in mice to overcome mitochondria heteroplasmy by targeting and selectively destroying diseased mitochondria but still allowing for the transmission of wild-type mitochondria in mouse oocytes from the original mother [20].

#### CRISPR/Cas9

This system also consists of two components. First, the Cas9 protein is a nuclease. Second, the CRISPR/Cas9 system consists of a synthetic guide RNA (sgRNA) [21]. The sgRNA is used for sequence specificity and has a 20 bp target recognition domain. However, the sgRNA contains more information than just targeting specificity and has a complex stem loop structure. The loading of the sgRNA is critical for activating the catalytic activity of Cas9 [22]. The binding and catalytic activity of the Cas9/sgRNA complex on target DNA is also dependent on the presence of an external sequence known as the Protospacer Adjacent Motif (PAM) [23]. Target DNA sequences complementary to the sgRNA are ignored by the Cas9/sgRNA complex if PAM is not present upstream of the target DNA. This is because DNA strand separation and the RNA–DNA heteroduplex are initiated at the PAM site (Fig. 1c). While there are target sequence limitations created by the requirement of PAM before the target sequence, research into overcoming the initial limitations is showing that by using Cas9 orthologs with alternative PAM sequences [24–26] and distinct sgRNA recognition sequences [27] there may be no practical limit to the targetable sites in the genome. Another practical advantage of CRISPR/Cas9 is the relative ease of creating short custom nucleotide (<100 bp) sequences compared to engineering proteins to bind different sequences while also maintaining activity and specificity. The CRISPR/Cas9 protocol [28–32] has undergone numerous improvements, allowing it to become the most widely used genome editing system. The advantage of CRISPR/Cas9 over ZFNs and/or TALENs is its scalability in that multiple sites within the mammalian genome can be modified simultaneously, providing a robust, high-throughput approach for gene editing in mammals. These advantages in this system are largely due to the fact that RNA, instead of designed protein, is used to specify the target.

The CRISPR system has been adapted to target double-strand breaks and modify DNA sequence in the genomes of a number of organisms including humans [33], mice [34], and zebrafish [35]. In fact CRISPR/Cas9 has been adopted to work in species ranging from prokaryote to eukaryote, plant to animal, and vertebrate to invertebrate. Of particular interest is the fact that in its earliest stages of development CRISPR/Cas9 has been used to alter the mouse by using a knockin system [36, 37] and also enabled reverse genetic studies in post-mitotic neurons of the adult brain [38]. CRISPR/Cas9 has also been used to generate one-cell embryos that go on to develop into edited monkeys [39]. Finally, it has been demonstrated to deplete synaptic proteins in rat hippocampal neuron cultures [40] and correct the mutation in the hemoglobin beta gene responsible for sickle cell anemia [41]. This was done in blood cells derived from induced pluripotent stem cells (iPSCs) of patients and with a higher efficiency than possible using ZFNs and TALENs [41, 42].

The CRISPR/Cas9 system can also be used to create gene drives [43]. Gene drives are a synthetic biological system in which a transgene can aggressively propagate independently of natural selection. It can quickly take over a population in a few generations with only just a few founders. This is because the mutation edited into a founder will overwrite the content of the wild-type parent in their offspring, thus it overcomes the diploid genome and makes an inherited heterozygote and homozygote [44].

### Genome editing the epigenome

Genome editing systems can also be used to edit the epigenome in a fashion that is distinct from epigenome editing, as it involves altering sequence critical to the epigenome. CRISPR/Cas9 has been utilized to study chromatin architecture and make targeted and unprecedented alterations to the repeat rich regulatory elements. It created deletions, inversions, and duplications that enabled the study of the clustered protocadherins [45], a complex locus that generates individual neuronal identity and is involved in neurodevelopmental disorders [46]. The approach has lead to the discovery of regulatory elements from the protocadherin α cluster that are also involved in the regulation of the γ cluster. Finally, CRISPR/Cas9 has been used to remove CTCF binding sites in the Hox gene clusters during embryonic stem cell differentiation in cervical motor neurons and disrupt the topological chromatin boundaries, turning repressed chromatin into active chromatin by preventing CTCFs targeted function as a genomic insulator [47]. Also, TALENs have been used to study long-range chromatin interactions by altering the sequence of inter- and intra-chromosomal contact points [48].

Furthermore, the cross-species capability of CRISPR/Cas9 has lead to profound insight in mammalian systems that were previously inaccessible at the level of basic research, particularly in monkeys [49] and humans. CRISPR/Cas9 has already been used to investigate DNA methylation machinery. Two genome editing approaches were developed in an in vivo mouse model, one had a single gene approach to target the reader *MeCP2* that allowed for visualization and cell sorting and the other had a multi-gene approach to target the DNA methyltransferases (DNMTs) *Dnmt1*, *Dnmt3a*, and *Dnmt3b* [38]. More recently, the CRISPR/Cas9 system was used for experimentation in human embryonic stem cells to create precise knockout deletions in the DNMTs [50]. By creating catalytically inactivating mutations using both multiplex and singleplex approaches the targets of DNMT1, DNMT3A, and DNMT3B were mapped with single-base pair resolution via whole-genome sodium bisulfite sequencing. This study was done in reference to a previous mouse model study of embryonic stem cells [51]. In both humans and mice, ESCs are viable without DNMT3A or DNMT3B, but interestingly only human cells undergo rapid cell death from the removal of DNMT1. This occurred even with an inducible system to control the temporal disruption, where cell death occurs immediately upon inducing repression of the system masking the homozygous mutation. Thus, the CRISPR/Cas9 technology was able to illustrate a fundamental difference between humans and mice.

### Epigenome editing

Besides editing genome sequence, genome editing systems have been used in altering the transcription of specific gene(s) without altering the underlying sequence. This modification for transcriptional alterations involves exclusion or inactivation of nuclease activity followed by use of the targeting system fused to a modular effector domain and is known as epigenome editing. A list of effectors and their effects on gene expression (increase or decrease) is summarized in Table 1. However, it should be noted that the effect of epigenetic marks is context dependent and thus the context of this table is the average consequence of depositing these marks in a promoter or enhancer. These systems have been used to target integrated/synthetic as well as endogenous loci, a distinction that is reviewed by de Groote et al. [1].

**Table 1:** A comprehensive selection of effector domains for epigenome editing systems

Effect	Domain	Consequence	Reference(s)
Activation	VP64 VP16 p65 SAM VPR	Activates transcription by recruiting a transcription complex and can then recruit histone acetylation as a consequence of the induced transcription. Can recruit p300 to deposit H3K27ac	Seipel et al. [52] Beerli et al. [53] Konermann et al. [57, 84] Gilbert et al. [81, 87] Mali et al. [83] Perez-Pinera et al. [30] Kearns et al. [85] Hu et al. [95] Tanenbaum et al. [96] Gao et al. [54] Chakraborty et al. [90] Heller et al. [125] Zalatan et al. [88] Nihongaki et al. [102] Polstein and Gersbach [103] Chavez et al. [97]
Repression	KRAB SID Tbx3-RD	Represses transcription by preventing transcription complex formation. May also recruit other repressive modifications	Margolin et al. [55] Ayer et al. [56] Cong et al. [17] Gilbert et al. [81, 87] Chen et al. [105] Kearns et al. [85] Hu et al. [95] Ma et al. [59] Gao et al. [54] Zalatan et al. [88] Telese et al. [89]
DNA methylation	DNMT3A DNMT3B M.EcoHK31I M.HhaI M.SssI DNMT3A-3L	Represses transcription	Li et al. [63] Meister et al. [64] Chaikind et al. [66, 69] Rivenbark et al. [65] Siddique et al. [67] Nunna et al. [68] Stolzenburg et al. [70] Kungulovski et al. [71] Bernstein et al. [76]
DNA demethylation	TDG TET1 TET2	Activates transcription	Gregory et al. [72] Maeder et al. [73] Chen et al. [78]
H3K9me	G9a (EHMT) Suvar KYP GLP	Represses transcription	Snowden et al. [61] Falahi et al. [62] Konermann et al. [57] Heller et al. [125] Kungulovski et al. [71] Cho et al. [74]
H3K9 demethylation	JMJD2B	Activates transcription	Hu et al. [95]
H3K9 deacetylation	Sin3a	Represses transcription	Konermann et al. [57]
H3K4me2 demethylation	LSD1	Also leads to H3K27ac removal; both repress transcription. Ideal for enhancers	Mendenhall et al. [75] Kearns et al. [58]
H3K27me3	NUE	Represses transcription	Konermann et al. [57]
H3K27 demethylation	JMJD3	Activates transcription	Hu et al. [95]
H3K27ac	p300	Activates transcription. Ideal for enhancers	Hu et al. [95] Hilton et al. [91]
H4K8 deacetylation	HDAC8 RPD3 Sir2a	Represses transcription	Konermann et al. [57]
H4K20me3	tgSET8	Represses transcription	Konermann et al. [57]
Affinity enrichment	PrA FLAG	Locus-specific chromatin enrichment for protein analysis	Byrum et al. [109, 113] Fujita et al. [110, 111] Waldrip et al. [112]
Cell imaging	GFP Cherry BFP	Sub-nuclear visualization	Chen et al. [46] Anton et al. [106] Ma et al. [108]

The function of ATF was the primary purpose for DNA sequence specificity of designer ZFs and TALEs with the goal of precise transcriptional activation, also known as transactivation. Transactivation effector domains are based on viral elements. The original VP16 domain [52] comes from Herpes Simplex Viral Protein 16 and consists of amino acids 437–447 [DALDDFDLDML]. VP16 was later engineered into VP64 domain [53], which is a fusion containing four tandem copies of VP16 connected by glycine-serine linkers [DALDDFDLDML]-GS-[DALDDFDLDML]-GS-[DALDDFDLDML]-GS-[DALDDFDLDML]. It is the most widely used transactivation domain. Interestingly, one effect of using the VP64 transactivation domain is that it recruits p300, which causes activating H3K27Ac to increase at the targeted locus over time and represents an example of transcription driving transcription [54]. Transcriptional repression, on the other hand, utilizes repression domains [55, 56] and is typically achieved by variants of a 45-aa segment from Krüppel-associated boxes (KRAB) [55] or repressive epigenomic modifications [57, 58]. The KRAB repressor domain appears to be the most potent natural repressor in the genome and used by half of zinc fingers, which make up half of the genome’s transcription factors. Interestingly, the KRAB repressor domain recruits histone modifying domains and results in a decrease of activating H3K4me3 and increase of repressive H3K9me3 and H3K27me2 but does not alter DNA methylation [59]. However, these modifications may not reflect the immediate effect of transcriptional repression and could be a later consequence [60]. A bacterial DNA methyltransferase (M.SssI) is also capable of repression and recruiting a heterochromatin protein, H3K9me3, and H3K27me2 [59]. The following are examples of how some of these effector domains have been used with DNA-binding platforms to modulate gene expression.

#### Zinc Fingers

The ZF system has been extensively used as an artificial transcription factor (Fig. 1d). It was first used to establish epigenome editing in 2002 when an engineered ZF fused to a histone methyltransferase was able to show that H3K9 methylation is causative in gene repression [61]. Since then ZFs have been designed with histone methyltransferases to repress oncogenes [62]. ZFs have also been utilized with DNA methylation machinery. In these cases, the DNA methyltransferases (DNMTs) were fused to designer ZFs to cause targeted DNA methylation and repress related gene expression [63–70]. Such designs have varied from engineered bacterial methyltransferases to select domains of the mammalian DNMT family. Recently, a ZF fused to DNMT3A or the H3K9 methylation writer GLP were delivered by adenoviral delivery system to control the regulation of a cancer gene by targeting its promoter [71]. While DNA methylation repressed longer than H3K9 methylation, the effect was not long-lasting and the authors speculate that multivalent epigenetic modifications must be designed for long-term effects when epigenome editing and to accommodate for large-scale chromatin domains. One promising design of effector domain involves a fusion of the catalytic domain of the de novo methyltransferase DNMT3A and C-terminal domain of (the catalytically inactive) DNMT3L, which naturally stimulates DNMT3A’s activity [67]. Alternatively, ZFs can be used to enhance gene expression by being fused with the DNA demethylase thymidine DNA glycosylase (TDG) [72]. However, epigenetic editing by ZFs is prone to the same problems as genome editing, genome wide off-target effects caused by the nature of ZF recognition being altered by additional (epi)genomic context [60].

#### TALEs

Taking the modular approach of ZFs, TALEs have been modified to induce transcriptional activation [73] and repression [74] (Fig. 1e). TALE epigenome editing systems have been used to target and modify chromatin at enhancers [75] and regulate gene expression via DNA methylation [76]. Furthermore, using a combination of epigenome editing systems and optogenetics for light induction, it was shown that gene expression, histone acetylation (H3K9ac) and histone methylation (H3K27me3) in the mouse brain can be targeted and modulated in a reversible fashion [57] at will. TALEs have also been efficiently fused to the TET family of active DNA demethylases and drive gene expression in targeted sequences by actively removing DNA methylation [77, 78]. The fusion of the DNMT3A and DNMT3L was replicated in TALEs [76].

#### dCas9

The CRISPR/Cas9 system has also been used to alter transcription [79–88]. This has been achieved by targeting with sgRNAs and a catalytically inactivated Cas9 (dCas9), which creates a RNA-based targeting system that can be fused to effector domains. Therefore, dCas9 based epigenetic editing gains the target specificity of CRISPR/Cas9 without causing a double-stranded break and while carrying out the function of the effector domain at the target site (Fig. 1f). A dCas9 system fused to the KRAB transcription repressing domain has been used to confirm the function of LRP8-Reelin-regulated neuronal enhancers in cortical neurons and lead to the discovery of a novel synapse-to-nucleus pathway related to glutamatergic signaling [89]. However, in the context of transcriptional control the dCas9 system often achieves low effectiveness, which can be improved by the tiling of multiple sgRNAs. Yet another approach to transcription activation via dCas9 has been to fuse two activation domains per dCas9. This system was used to reprogram the cell lineage of stem cells and drive subsequent phenotypes [90]. Also, the histone acetyltransferase p300 can be used as an effector domain to achieve H3K27 acetylation and induce gene expression by targeting the mammalian β-globin locus control region, which is something that could not be achieved by a VP64 domain [91].

Since each system has its own strengths and weaknesses when it comes to off-target and on-target effects, price, cellular toxicity, and ease of use, a standardized comparison system is needed [92, 93]. In practice, each system may show a unique potential when used on their own or coupled together [94]. This has recently been exemplified in the case of transcriptional activation of pluripotency factors in humans and mice by both CRISPR- and TALE-based editing systems [95]. When comparing the ability to drive gene expression by targeting enhancers, it was found that TALEs could outperform CRISPR/Cas9 and the authors recommended an approach combining both systems for highly efficient transcriptional regulation [54]. However, this comparison used initial and less effective CRISPR activators and not the most enhanced genetically engineered improvements [84, 87] that are described below.

### Engineered improvements

**Fig. 2:**
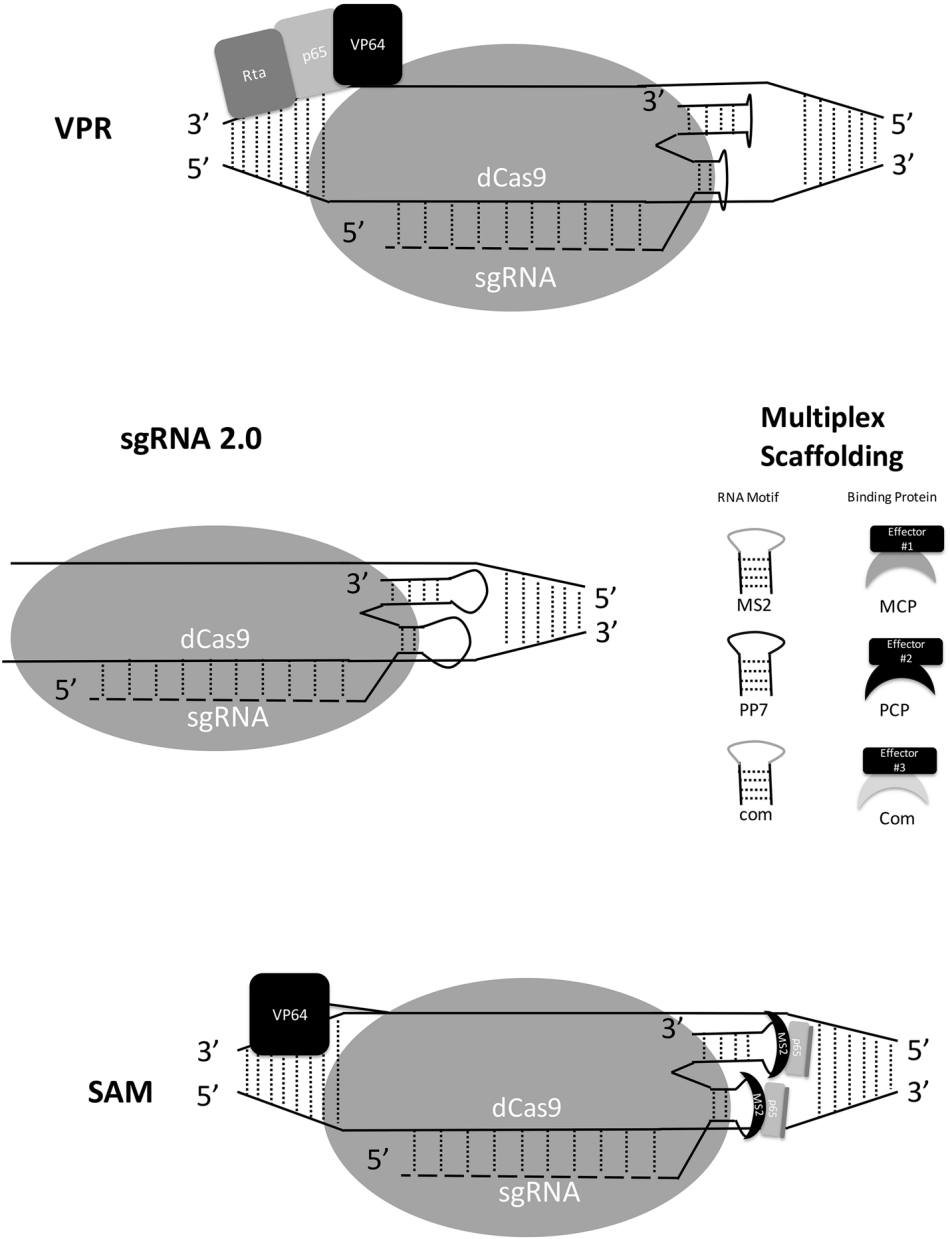
Designer epigenome editing systems based on dCas9. VPR refers to the effector domain, which is a tripartite design. sgRNA 2.0 refers to a scaffolding system that allows for modular effectors to be added to the sgRNAs that have been modified to contain protein-binding sites for RNA recognizing proteins. SAM refers to a synergistic activator that contains an effector domain fused to the dCas9 protein as well as the sgRNA 2.0 design to add additional designer activators. Components are not to scale as critical features are exaggerated and some non-variable features are removed

Several laboratories have begun to utilize the genetically engineered Cas9 proteins and sgRNAs (Fig. 2). One alterative involves a versatile scaffolding platform to attach multiple VP64 domains to dCas9 [96] and a different approach uses the tripartite activator VP64-p65-Rta (VPR) [97]. Another variation is the use of second-generation sgRNAs (sgRNA 2.0) for multi-effector programming [88], where scRNAs (extended sgRNAs) have effector domain recruitment sites added into their sequence after the targeting site. This approach further creates modularity in that there are layers of variation created in Cas9 orthologs having different sgRNA recognition sites. Additional layers are then created by the fact that each sgRNA can be programmed to have two recognizable loops that can be bound in homogenous or heterogeneous configurations by a unique binding protein being fused to unique effector domains [88]. One example of this system is the Synergistic Activation Mediator (SAM), which is a potent transcription activation system [84]. In SAM, the exposed and engineered RNA loops from the Cas9–sgRNA–DNA complex are used as anchoring points for the RNA-binding protein (MS2) that is also fused to a p65-HSF1 fusion effector domain. This allows for a synergistic combination in activating gene expression at levels much higher than a single effector domain. Another approach involves further enhancing the sgRNA to create a system known as CRISPR-Display [98]. CRISPR-Display allows for functional RNA domains (~4.8 kb) to be inserted into the sgRNA loops at multiple positions, including the same loop as sgRNA 2.0 as well as 5′ and 3′ positions. This approach uses functional motifs like the protein-binding cassettes of earlier sgRNA 2.0 approaches but also enables long non-coding RNA to be inserted into the dCas9/sgRNA complex. Ultimately, the CRISPR-Display system enables precision ectopic targeting of RNA and ribonucleoprotein to loci of interest in order to fully characterize the functionality of the RNA.

### Combinatorial biotechnology

(Epi)genome editing systems allow not only for fusing a genome editing system to an effector but they can also add additional genetic changes and facilitating methodologies. For example, Konermann et al. [57] used transcriptional control or histone modifying (acetylation and methylation) effector domains (Table 1) along with a light inducible (optogenetic) element. The optogenetic induction system involves light-sensitive cryptochrome 2 (CRY2) and CIB1, its binding partner [99]. These two proteins only heterodimerize upon exposure to blue light, a process that is rapid and reversible, and can be applied to study neurons in mammalian brains [100, 101]. They can then be separately fused to an epigenome editing system, with one attached to the targeting system and the other to the effector. This can be utilized in epigenome editing by allowing for the editing to be induced when and where it is desired in a rapid and reversible manner. The optogenetic approach has also been combined with CRISPR/Cas9 transactivation systems [102, 103]. Alternate inducible transactivation systems use steroids that has been created using TALEs [104]. Finally, cell imaging can also be achieved using an EGFP effector domain [105] that can visualize pericentric, centric, and telomeric repeats [106]. This allows for the visualization of repetitive sequences using a single sgRNA or an array of sgRNAs for non-repetitive sequences to enable visualization and tracking through cellular processes. This technique was demonstrated by imaging telomere dynamics and the dynamic sub-nuclear localization of a single gene through mitosis. Another visualization system has been developed that allows for multicolor analysis. It was initially successful in TALEs [107] and was recently adopted for dCas9 orthologs with three spectral systems [108]. The spectral systems were used to target telomeres, several target loci, and also determine the intranuclear distance between loci on different chromosomes, which allowed for the assessment of DNA compaction in live cells.

Furthermore, epigenome editing systems have been used for enrichment and purification of proteins interacting with target loci. They have the potential to allow for an examination of all the proteins and histone PTMs associated with a single genomic locus, the epiproteome [109]. These techniques couple chromatin immunoprecipitation and the target specificity of genome editing systems (without the catalytic activity) by using an effector domain to allow for enrichment that can then be coupled to analysis by mass spectrometry. One variant of this approach is known as engineered DNA-binding molecule-mediated chromatin immunoprecipitation (enChIP). It has been developed using TALEs [110] and CRISPR/Cas9 [111] to study telomeres. Another variant involving the CRISPR/Cas9 system has also been developed and termed Chromatin Affinity Purification with Mass Spectrometry (CRISPR-ChAP-MS) [112]. This approach was able to reveal the changes during the activation of transcription although it had difficulty with repressive contexts, including when using TALEs [113]. The developments identified above represent some selected issues that will be further enabled using epigenome editing technology. More importantly, we anticipate additional future modifications and applications of this system to provide insights into other biological problems that have remained difficult to investigate.

### Future challenges

A flurry of publications in recent years have established that (epi)genome editing may hold the key to the next generation of genomic revolution; the alteration of gene sequences as well as its expression in designated tissues at will. To date, most of this research has focused on understanding biological complexities including correction of gene defects that may be associated with diseases. It is also apparent that genome editing systems have met a number of challenges in providing the needed proof of principle for epigenome editing systems. These include off-target effects, editing efficiency, delivery [114–116], and cytotoxicity [117]. In particular given the complexity of the genome sequence and organization, it may not be easy to avoid off-target effects in most if not all cases, particularly in the highly open and dynamic chromatin of embryos that is not well characterized. These limitations mean that only some of the cells in question will have the desired outcome. This level of correction and expected somatic mosaicism may be sufficient in some but not all cases.

It is apparent that most research on genomic correction in humans will involve ex vivo methods [118]. The ex vivo approach involves harvesting appropriate cells from the patient, correcting them in culture, and then returning the corrected cells via autologous transformation. The in vivo approach involves directly transforming somatic cells in the patient. On the other hand, ZFs can cross cell membranes and induce genome editing in human cells [119, 120]. Furthermore, incorporating tandem nuclear localization signal repeats into the ZFN protein backbone may improve cell permeability to ~13-fold and allow for genome modification success rates of 26 % in CD4+ T cells and 17 % in CD34+ hematopoietic progenitor cells [121]. TALENs can also be modified for enhanced cell penetrating abilities by conjugating with peptides that allow for optimized protein machinery delivery. It may allow for effective parallel viral transfection [122]. Cas9 and sgRNAs have also been utilized for effective genome editing that does not require transformation of the editing system into host by using common cationic lipid nucleic acid transfection reagents to deliver the system [123] or by using electroporation [124]. As it stands, there are still key obstacles to overcome with epigenome editing but given the exponential rate of advancement most technological limitations will shortly be overcome.

## Conclusion

It is apparent from the examples listed above that future application of epigenetic correction using the current and evolving technologies is only limited by imagination. Besides monogenic diseases, epigenome editing may apply in cases of complex traits, such as the long-term effects on neuroplasticity from stress and drug exposure. A recent example showed that a locus-specific epigenetic remodeling may control cocaine addiction- and depression-related behaviors [125]. This study used ZFs and TALEs to target histone methylation (H3K9me2 via G9a) or acetylation (correlated with transcriptional activator; p65) at transcription factor binding sites (SRF and CREB) of the *Fosb* promoter in the nucleus accumbens, a brain region involved in reward and addiction.

There is every reason to argue that cell-type-specific epigenome editing systems should be used to modify cells in vivo and/or in vitro to further basic science as well as correct a variety of diseases. Ultimately, epigenome editing represents a much-needed tool for the advancement of functional genomics and personalized medicine. Yet, until the trans-generational and population level consequences are fully understood and debated it must remain limited to somatic cells and not cross to the human germ-line.

## Abbreviations

ncRNA: non-coding RNA
DNMT: DNA MethylTransferase
5mC: 5-MethylCtyosine
5hmC: 5-HydroxyMethylCtyosine
PTM: post-translational modification
ZF (or ZFP): Zinc Finger (Protein)
ZFN: Zinc Finger Nuclease
TALE: Transcription-Activator-Like Effector
TALEN: TALE + Nuclease
RVD: repeat variable di-residue
CRISPR/Cas9: Clustered Regulatory Interspaced Short Palindromic Repeats (CRISPR)-Associated 9 (Cas9)
sgRNA: short guide RNA
PAM: Protospacer Adjacent Motif
ATF: artificial transcription factor
TDG: thymidine DNA glycosylase (DNA Demethylase)
TET: Ten-Eleven Translocation (DNA Demethylase)
dCas9: deactivated Cas9
VPR: VP64-p65-Rta
scRNA: scaffolding RNA (sgRNA 2.0)
SAM: Synergistic Activation Mediator
